# Synergistic Effects of Biochar and *Bacillus thuringiensis* NL-11 on *Ophiopogon japonicus* Growth and Soil Microbial Diversity in Trampled Urban Forest Soils

**DOI:** 10.3390/microorganisms13092004

**Published:** 2025-08-27

**Authors:** Qianqian Liu, Hui Nie, Xiaorui Sun, Lina Dong, Liu Xiang, Jinchi Zhang, Xin Liu

**Affiliations:** 1 Co-Innovation Center for Sustainable Forestry in Southern China, Jiangsu Province Key Laboratory of Soil and Water Conservation and Ecological Restoration, Nanjing Forestry University, Nanjing 210037, China; lqq77@njfu.edu.cn (Q.L.); nh1263179511@njfu.edu.cn (H.N.); taylorrui240@njfu.edu.cn (X.S.); liuxiang7386@njfu.edu.cn (L.X.); 2 Zhongshan Cemetery Administration, Nanjing 210037, China; donglina90@sohu.com

**Keywords:** urban forest, soil restoration, *Bacillus thuringiensis* NL-11 application, biochar, plant growth

## Abstract

Bare soil expansion in urban forests, driven by persistent high-intensity trampling, degrades both macro-scale natural resources and micro ecological conditions. Targeted interventions are therefore essential. In this study, trampled bare ground in forest parks and artificially cultivated *Ophiopogon japonicus* were used as experimental models We employed trampled bare ground in forest parks as well as artificially cultivated *O. japonicus* as experimental models. Five treatments were implemented: enclosure control (CK), ploughing (F), *Bacillus thuringiensis* NL-11 application (J), biochar addition (C), and co-application of *B. thuringiensis* NL-11 with biochar (JC). Our results indicate that, compared with CK, biochar treatments reduced soil bulk density by 30%, increased soil porosity by 89%, and improved water-holding capacity. The soil nitrate nitrogen content in the NL-11 treatment was increased by 113.8% compared with CK, while the co-application of NL-11 with biochar exhibited the highest sucrase and urease activities. Notably, the co-application of *B. thuringiensis* NL-11 with biochar exhibited the most pronounced effects on aboveground biomass, plant height, and root development, followed by the *B*. *thuringiensis* NL-11 treatment. Microbial β-diversity shifts under co-application of *B. thuringiensis* NL-11 with biochar treatment strongly correlated with soil enzyme activation and plant growth enhancement (Mantel test, *p* < 0.05). Correlation analysis confirmed that exogenous nutrient inputs significantly influenced enzyme activities, thereby promoting plant development. These results highlight the effectiveness of integrating microbial inoculation with biochar to restore trampled urban forest soils.

## 1. Introduction

Urban forests, as integral components of urban ecosystems, possess unique ecological, social, and economic values, which provide comprehensive ecosystem services and contribute significantly to urban sustainability and the enhancement of human well-being [1,2,3]. However, with the incessant acceleration of urbanization, human activities in urban areas are causing increasingly severe environmental problems that primarily manifested as the degradation of forest ecosystems [4,5,6,7]. Prolonged anthropogenic disturbances (e.g., trampling) result in the continued degradation of vegetation to bare ground, which leads to barren soil, reduced overall vegetation cover, and soil erosion [2,8]. Currently, trampling disturbances have emerged as a significant challenge for many urban forests [9,10]. Unregulated, intense trampling activities induce serious damage to forest soils and surface vegetation [11], and significantly impact both the macro natural resources and micro ecological environments of urban forest parks [12]. Trampling-induced soil compaction reduces pore connectivity, air permeability, and rooting space, hindering plant growth. Prolonged trampling drives bare land expansion, reducing plant diversity, disrupting soil systems [10], and causing soil erosion with habitat fragmentation [2,13,14]. Such degradation depletes nutrients (e.g., nitrogen, phosphorus, and potassium), increases surface runoff, hampers soil microbial communities and affects microbial-mediated soil biochemical reactions and ecosystem functions [15,16,17,18].

Therefore, in order to address the environmental problems caused by stampedes, some remedial measures must be taken. Phytoremediation avoids secondary pollution and demonstrates promising potential for restoring trampled bare lands [19]. Fencing for regeneration, which is one of the most commonly used restoration techniques for restoring degraded ecosystems, has been extensively applied in trampling restoration [20,21]. However, for trampled bare land that is characterized by high compaction and poor permeability, the effectiveness of short-term fencing remains limited. Ploughing is another prevalent soil restoration techniques that enhances soil permeability and accelerates soil matter cycling, thereby improving soil fertility [22], and can improve compaction of degraded urban soils [23].

Compared with traditional remediation methods, the synergistic biochar–microbial inoculant remediation technology, which enhances soil resilience through biochar and enables contaminant co-remediation, has been widely validated in field applications [24,25]. Biochar is a carbon-rich material formed through the pyrolysis of crop residues under oxygen-limited conditions, which is characterized by its porous structure and large surface area [26]. The application of biochar to soil has positive effects on the soil environment, including the provision of nutrients and stabilization of soil structures [27,28], and it can also improve the drought resistance of plants in urban soils [29]. On one hand, biochar can increase soil porosity and reduce bulk density, thereby enhancing soil water retention [30]. Correspondingly, its positive impact on the activities of soil enzymes is also a response to improved soil quality, nutrient utilization and microbial communities [30,31], and would be most pronounced in the most degraded soils [32].

Microbial inoculants (as a means of inducing microbial fertility) have been shown to protect soil structures, enhance soil fertility, improve plant stress resistance, and promote plant growth [33]. Our previous research has shown that adding plant rhizosphere-promoting bacteria, such as *Bacillus thuringiensis* NL-11, to the soil can enhance soil nutrients and improve soil structure [24,34,35]. Recent studies have suggested that certain combinations of biochar and microbial inoculants show significant promise in addressing common soil limitations on tree performance in urban settings [36,37], but it has also been noted that there may be no additional benefits when these materials are used in combination [38]. Restoring microbial diversity underpins ecosystem functional stability [39]. During restoration of trampling-degraded ecosystems, microorganisms play a crucial role in regulating material cycles, improving soil microenvironments, and promoting plant symbiosis to facilitate ecosystem recovery [40]. Therefore, it is necessary to study the impact of trampling restoration measures on soil microbial communities. However, current studies on the effects of their synergistic application on severely compacted soils caused by trampling in urban forests remain poorly understood. Therefore, this study investigated five restoration measures, namely enclosure (CK), ploughing (F), addition of microbial inoculants (J), addition of biochar (C), and combined addition of biochar and microbial inoculants (JC), on the effects on soil nutrients, enzyme activities, soil microbial communities and plant growth. This study focused on the urban forest soils of Zijin Mountain National Urban Forest Park, which have been subjected to long-term trampling disturbances and are unlikely to recover naturally. This study hypothesizes the following: (1) These restoration measures can improve the soil condition and promote plant growth. (2) The combined application of biochar and microbial inoculants showed the most significant improvement in the soil environment and plant growth among all the remediation measures.

## 2. Materials and Methods

### 2.1. Site Description and Experimental Design

**Figure 1:**
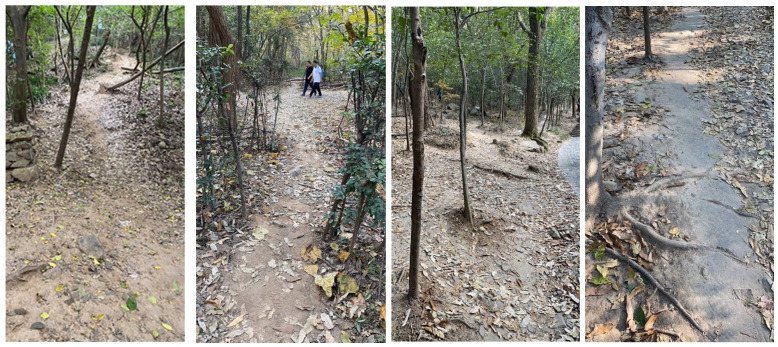
Sections of trampled paths in Zijin mountain urban forest park.

Zijin Mountain National Urban Forest Park, located in the northeastern portion of Xuanwu District, Nanjing, Jiangsu Province, China (118°81′–118°88′ E, 32°04′–32°09′ N), has an elevation of 448.8 m. Characterized by a subtropical monsoon climate, it experiences an average annual precipitation of 1530.1 mm and average annual temperature of 19.6 °C, with a frost-free period of 322 days. The experimental site is situated on the southern slope of Zijin Mountain with terrain that is dominated by low hills, which was primarily developed from Jurassic gravelly quartz sandstone, resulting in yellow brown soil. The soil pH ranges from 4.8 to 6.0, is primarily a sandy loam in texture, with a soil bulk density of from between 1.35 and 1.65 g/cm^3^. The forest coverage rate is 70.2%, with dominant tree species that include *Quercus acutissima*, *Liquidambar formosana*, *Robinia pseudoacacia*, and *Ligustrum lucidum*. The primary vegetation communities are mixed coniferous and broadleaf forests, deciduous broadleaf forests, and mixed deciduous and evergreen broadleaf forests, along with some artificial bamboo forests, which were mostly formed by years of natural succession. This park shows obvious signs of human disturbances, which have created several elongated forest gaps. The understory includes over 300 bare pathways and patches of varying sizes formed by decades of human trampling, leading to ecological issues such as reduced biodiversity and soil erosion (Figure 1).

### 2.2. Experimental Materials

#### 2.2.1. *Bacillus thuringiensis* NL-11 Preparation

*Bacillus thuringiensis* NL-11 was isolated from the soil around a weathered dolomite [41]. Studies have demonstrated the suitability of microbial inoculants for soil improvement [42]. For this study, application of *B. thuringiensis* NL-11 was selected because it is capable of forming dominant microbial communities and can provide the soil with more readily available nutrients, thereby promoting plant growth [41,43]. The bacterium was introduced into a liquid medium and fermented in a shaker for 24 h. Subsequently, the microbial liquid was transferred to a fermenter, and the fermentation was considered complete when the optical density at 600 nm (OD_600_) reached its peak. The culture was then transferred to a sterile container and stored in a refrigerator.

#### 2.2.2. Biochar

The selected biomass char was derived from straw (SC-101 type), which was procured from Pingdingshan Tanuo Environmental Material Co., Ltd (Pingdingshan, China). Produced from straw at 550–600 °C under oxygen-free, nitrogen-protected pyrolysis, this biochar exhibits a neutral to slightly alkaline pH (7–7.5), high carbon content (≥95%), low ash content (5–10%), fine particle size (100 mesh), and a high specific surface area (900–1300 m^2^/g). Detailed introduction is shown in the Table S1.

#### 2.2.3. *O. japonicus*

The *Ophiopogon japonicas (L. f.)* Ker Gawl. is an important herbaceous plant in garden landscaping. It is a perennial tuft-forming herbaceous plant with creeping stolons, particularly extensively utilized in urban forests. It exhibits notable shade tolerance and drought resistance, which characterize its robust stress resilience [44]. Concurrently, it has specific soil requirements; the bulk density of the planting soil influences the emergence and growth of its fibrous roots, while the nutrient retention and water-holding capacities of the soil significantly affect plant growth [45]. The *O. japonicus* specimens were meticulously sourced from the seedling base of Zhongshan Nursery Farm in Nanjing. These specimens exhibited a pristine surface, devoid of disease and mechanical damage, with a consistent plant height ranging from 20 to 25 cm. The careful selection and sourcing of *O. japonicus* specimens from a reputable nursery farm enhanced the reliability and consistency of the experimental materials, which contributed to the robustness of the study.

### 2.3. Experimental Design

Study on the Impacts of Artificial Ecological Restoration Measures on Trampled Soil Properties: This research was conducted in areas of severely trampled soil (predicted to be incapable of natural recovery) to evaluate the effectiveness of different ecological restoration strategies. The experiment incorporated five treatments (enclosure control, ploughing, *B. thuringiensis* NL-11 application, biochar, and ploughing combined with both *B. thuringiensis* NL-11 and biochar) with all plots enclosed for the duration of the study. A randomized block design was used, with each treatment plot measuring 3 m× 2 m and a minimum of 1 m spacing between plots. Simple partitions were erected between the plots, and each treatment was replicated nine times. The soil was uniformly tilled to a depth of 30 cm, whereafter biochar was applied at a rate of 4.8 kg/m^2^ and bacterium at 1.5 L/m^2^. The application of the five treatments was completed on 30 June 2021. *O. japonicus* was planted on 7 July 2021 at a transplant density of 12 plants/m^2^. Soil and plant samples were collected on 7 July 2022 for the determination of various indicators.

### 2.4. Sample Collection

#### 2.4.1. Plant Sampling

For each treatment group, five plants were randomly selected for sampling. The soil was removed from the root systems, and the entire plants were rinsed clean. After the removal of excess water the plant heights were measured. The root indices were determined using a root scanner.

#### 2.4.2. Soil Sampling

Soil samples were collected from nine locations per treatment following the removal of surface litter, twigs, and leaves. After passing the samples through a 2 mm sieve to remove surface detritus, gravel, and plant and animal remnants, the soil profiles were excavated using the standard ring method to assess the physical soil properties. The samples were then stored in sterile, sealed bags, with a portion stored at −80 °C pending the determination of ammonium nitrogen, nitrate nitrogen and microbial testing. Another portion of the soil sample was air-dried and sieved for the assessment of soil chemical properties and enzyme activities.

### 2.5. Determination Index and Method

#### 2.5.1. Plant Analysis

After cleaning, the root system was placed on a high transparency scanning tray and spread out maximally for scanning with a root scanner (WinRHIZO Tron, Regent Instruments Inc., Quebec, QC, Canada). Grayscale scanning was performed, and root morphology indices were analyzed using the WinRHIZO root analysis system. The aboveground and belowground portions of the plants were weighed separately, placed in envelopes, and subjected to a kill-green process at 105 °C for 30 min, followed by drying at 85 °C until a constant weight was achieved.

#### 2.5.2. Soil Physical Properties

The soil compaction was measured using a soil compaction meter JSD-A1. The soil physical properties such as minimum water-holding capacity, bulk density, maximum water-holding capacity, capillary water-holding capacity, non-capillary porosity, capillary porosity, and total porosity were determined following the ring knife method as Shidan Bao [46].

#### 2.5.3. Soil Chemical Properties

The soil pH was measured using a potentiometric method with a pH meter, maintaining a soil-to-water ratio of 2.5:1. The soil organic carbon was determined using the potassium dichromate oxidation external heating method with spectrophotometric measurement. The available phosphorus was determined using 0.05 mol/L hydrochloric acid-sulfuric acid extraction followed by molybdenum-antimony colorimetric analysis. Readily available potassium was measured using 1 mol/L ammonium acetate extraction and flame photometry. The total carbon and nitrogen in the soil were analyzed using an elemental analyzer (vario EL III). Soil ammonium nitrogen and nitrate nitrogen were determined using potassium sulphate extraction, followed by measurement with a continuous flow analyzer (SAKLAR SAN++/S-011300110537) [47].

#### 2.5.4. Soil Enzyme Activities

Sucrase, urease, acid phosphatase, and catalase activities were determined using standard methods. In brief, sucrase activity was assessed by incubating soil with sucrose solution at 37 °C; the reducing sugars released were quantified by the DNS colorimetric method, with absorbance read at 540 nm [48]. Urease activity was determined using urea as the substrate, incubated for 2 h at 37 °C, and the ammonium produced was measured spectrophotometrically at 660 nm [49]. Acid phosphatase activity were assessed using disodium ρ-nitrophenyl phosphate as the substrate, incubated for 1 h at 37 °C, and the ρ-nitrophenol produced was measured at 420 nm [50]. Catalase activity was quantified spectrophotometrically by reacting soil samples with hydrogen peroxide and measuring the decomposition products at the relevant absorption wavelength, following established protocols [51]. Specific operational procedures were followed according to the instructions provided in the reagent kit manual (Suzhou Keming Biotechnology Co., Ltd., Suzhou, China).

#### 2.5.5. DNA Extraction and Illumina Sequencing

Rhizospheric soil DNA was purified from 0.5 g aliquots using the FastDNA Spin Kit for Soil (MP Biomedicals, Irvine, CA, USA). DNA integrity was verified via 1% agarose gel electrophoresis, with quantification and purity assessment conducted on a NanoDrop 2000 spectrophotometer (Thermo Scientific, Waltham, MA, USA). Bacterial 16S rRNA gene fragments (V3–V4 region) were amplified using primer pair 338F (5′-ACTCCTACGGGAGGCAGCAG-3′) and 806R (5′-GGACTACHVGGGTWTCTAAT-3′) [52], while fungal ITS1 regions were targeted with primers ITS1F (5′-CTTGGTCATTTAGAGGAAGTAA-3′) and ITS2R (5′-GCTGCGTTCTTCATCGATGC-3′) [53]. Amplicons were carefully excised from 2% agarose gels and purified using an AxyPrep DNA Gel Extraction Kit (Axygen Biosciences, Union City, CA, USA) in strict accordance with the manufacturer’s instructions. The resulting purified amplicons were pooled at equimolar concentrations for paired-end sequencing (PE250) on an Illumina platform following standardized protocols. Sequencing data were processed via the UPARSE v7.1 software pipeline [54], with default UPARSE v7.1 software recommended parameters applied for the initial quality filtering of sequence reads. Post-filtering, paired forward and reverse reads were merged, chimera sequences were detected and removed, and the remaining reads were assigned to amplicon sequence variants (OTUs) based on identical nucleotide sequences. Chimera identification and removal were performed using the UCHIME algorithm [55]. After the elimination of chimeras, the denoised, chimera-free OTU sequences along with their corresponding abundances were exported. All raw sequencing reads have been archived in the NCBI Sequence Read Archive (SRA) under accession number PRJNA1262336.

### 2.6. Statistical Analysis

Before conducting statistical analyses, the dataset was evaluated for compliance with parametric assumptions: variance homogeneity was verified via Levene’s test, while normality was assessed through the Shapiro–Wilk W test implemented in SPSS 27 (Version 27.0, IBM Corp., Armonk, NY, USA). Parametric variables (normally distributed with equal variances) were analyzed using ANOVA to evaluate microbial treatment effects, whereas non-conforming datasets underwent non-parametric assessment via the Kruskal–Wallis test. Statistical analyses and graphical visualizations were executed in GraphPad Prism 9.5, with data represented as mean ± standard error (SE) (*n* = 9). Bacterial and fungal alpha diversity was assessed using the Abundance-based Coverage Estimator (ACE), Chao1, and Shannon index, which were calculated in R with the “vegan” R package (version 2.0-2) [56]. Graphic representations were created using Origin 2023 (Origin Lab Corporation, Northampton, MA, USA). The relationships between environmental factors and soil microbial communities were evaluated using Mantel tests and Pearson correlations with the R package “LinKET” (version 0.0.6.1) [57]. Constrained Principal Co-ordinates Analysis (CPCoA) analysis was generated in the R project “Vegan” package [57]. Hierarchical partitioning analyses were conducted via the “rdacca. p” R package (version 1.1-1) [58] to quantify the relative influence of environmental variables on soil microbial community composition and their associated ecological clusters.

## 3. Result and Analysis

### 3.1. Impacts of Ecological Restoration Measures on the Growth Characteristics of O. japonicas

**Figure 2:**
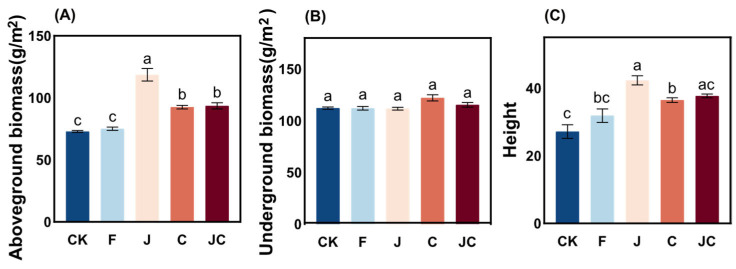
Effects of different restoration measures on growth characteristics of *O. japonicas.* Data were compared using Tukey-HSD test. (**A**): aboveground biomass, (**B**): underground biomass, (**C**): plant height. CK: enclosure control, F: ploughing, J: application of *B. thuringiensis* NL-11, C: application of biochar, JC: application of *B. thuringiensis* NL-11 + biochar; different lowercase letters showed significant differences between different treatments (*p* < 0.05).

Comparative analysis of ecological restoration measures revealed distinct effects on *O. japonicus* growth parameters (Figure 2A,C). Microbial inoculant (J) and biochar (C) elicited marked increases in aboveground biomass and plant height relative to the CK, with the J demonstrating optimal performance (*p* < 0.05). The C group showed the highest underground biomass among all groups, though non-significant (Figure 2B).

**Figure 3:**
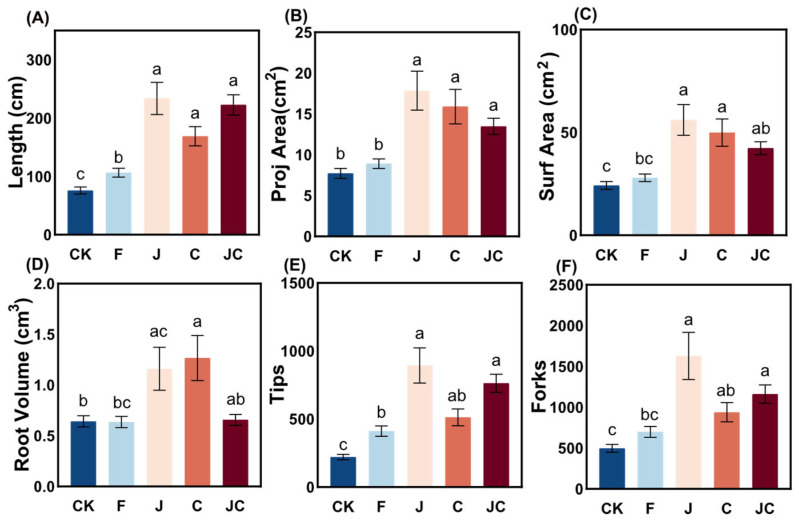
Influences of different restoration measures on the root morphology of *O. japonicas*. (**A**): length, (**B**): proj area, (**C**): surf area, (**D**): root volume, (**E**): tips, (**F**): forks; CK: enclosure control, F: ploughing, J: application of *B. thuringiensis* NL-11, C: application of biochar, JC: application of *B. thuringiensis* NL-11 + biochar; different lowercase letters showed significant differences between different treatments (*p* < 0.05).

The J and JC treatments showed significant increases in root architectural parameters relative to CK, including root length, projected area, surface area, root tips, and root forks (Figure 3) (*p* < 0.05). The J and C groups achieved superior root volume compared to CK (Figure 3D) (*p* < 0.05). Notably, the JC group exhibited lower root volume compared to both J and C groups.

### 3.2. Impacts of Ecological Restoration Measures on the Physicochemical Properties of Trampled Bare Soils

Restoration measures strongly impact key soil physical properties, including bulk density, water-holding capacity, and pore characteristics (Table S2). Compared with the CK, these restoration measures clearly reduced the soil bulk density while simultaneously increasing the soil water content and porosity. Among these, the C group exhibited significantly higher improvements than the other restoration measures in terms of these aspects (*p* < 0.05).

Specifically, the C group significantly reduced soil bulk density compared to other groups (*p* < 0.05). Furthermore, multiple soil hydrological properties including soil minimum moisture content, maximum moisture content, capillary moisture content, capillary porosity, and total porosity exhibited analogous patterns, with the C group demonstrating superior performance across all parameters and significantly higher values than CK (*p* < 0.05). The non-capillary porosities showed no significant variations across the various groups.

**Figure 4:**
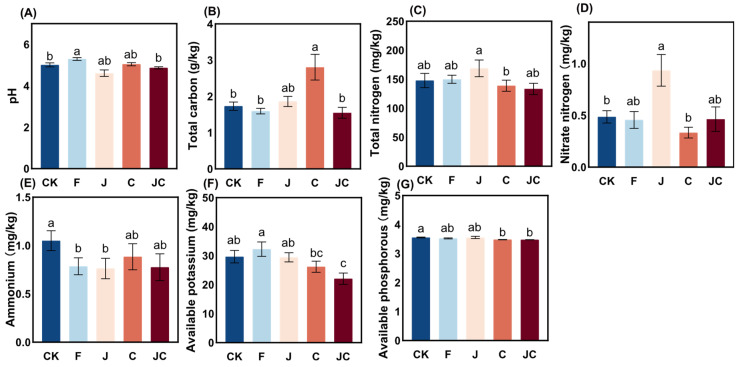
Impact trends of soil chemical properties under different ecological restoration measures. (**A**): pH; (**B**): total carton; (**C**): total nitrogen; (**D**): nitrate nitrogen; (**E**): ammonium; (**F**): available potassium; (**G**): available phosphorous; CK: enclosure control; F: ploughing; J: application of *B. thuringiensis* NL-11; C: application of biochar; JC: application of *B. thuringiensis* NL-11 + biochar. Different letters indicate significant differences between different restoration measures (*p* < 0.05).

Both J and JC groups exhibited numerically reduced soil pH compared to the CK, though these decreases lacked statistical significance (Figure 4A). The C group significantly elevated total carbon content relative to other groups (*p* < 0.05; Figure 4B). While the J group showed marginally higher total nitrogen and nitrate nitrogen (Figure 4C,D) than other groups, only nitrate nitrogen (Figure 4D) exhibited statistically significant elevation relative to the CK (*p* < 0.05). Conversely, soil ammonium nitrogen content (Figure 4E) was numerically greater in CK than the other groups, though non-significant. Notably, the JC group showed significantly reduced available potassium (Figure 4F) and available phosphorus (Figure 4G) concentrations compared to CK (*p* < 0.05), indicating concurrent depletion of these soil available nutrients.

### 3.3. Impacts of Ecological Restoration Measures on Enzyme Activities in Trampled Bare Soils

**Figure 5:**
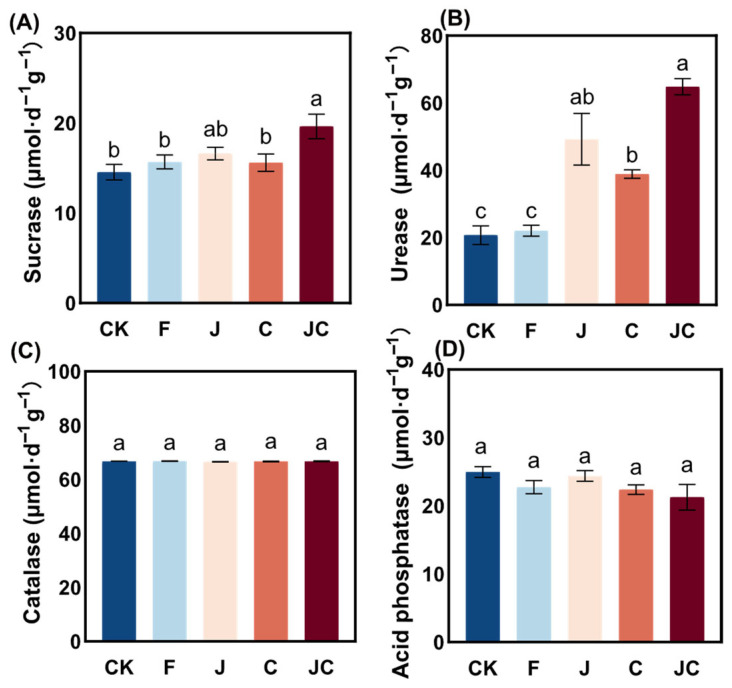
Impact trends of soil enzyme activities under different ecological restoration measures. (**A**): sucrase; (**B**): urease; (**C**): catalase; (**D**): acid phosphatase; CK: enclosure control; F: ploughing; J: application of *B. thuringiensis* NL-11; C: application of biochar; JC: application of *B. thuringiensis* NL-11 + biochar. Different letters indicate significant differences between various restoration treatments (*p* < 0.05).

Ecological restoration groups markedly altered enzyme activities in trampled bare soils (Figure 5). In particular, the activities of soil sucrase, urease, and catalase activities were highest under the JC group. Notably, the J and JC groups demonstrated significantly elevated soil urease and sucrase activities compared to the CK group (*p* < 0.05) (Figure 5A,B). The influences of restoration treatments, on soil catalase activity were negligible (Figure 5C,D).

### 3.4. Influence of Ecological Restoration Measures on Soil Microbial Diversity and Correlation

**Figure 6:**
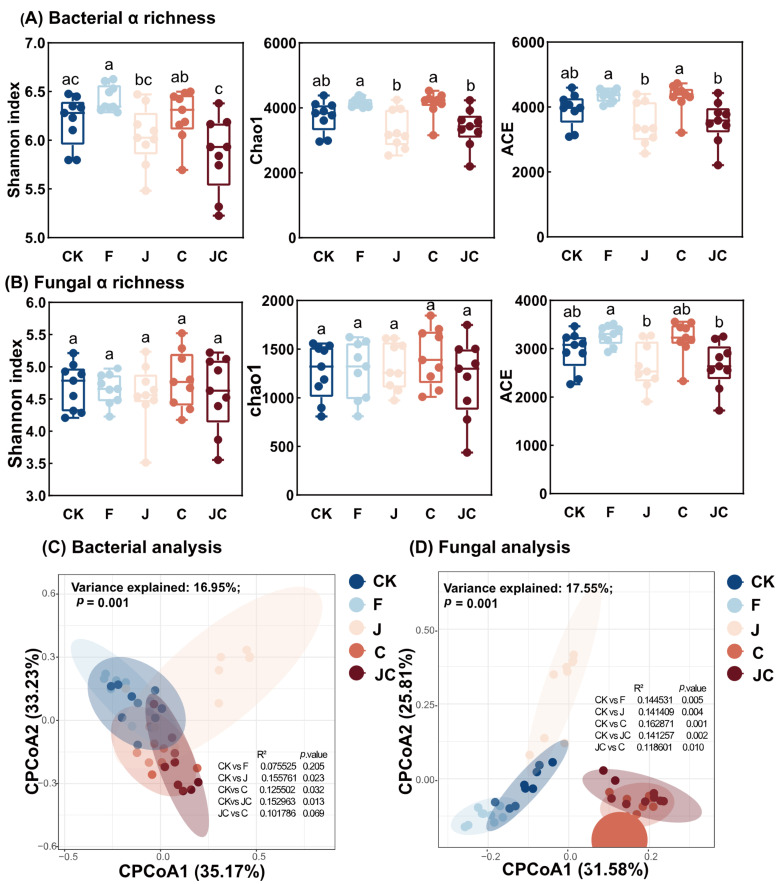
Effect of ecological restoration measures on bacterial (**A**) and fungal (**B**) α diversity. Different letters indicate significant differences for each treatment (*p* < 0.05). Bacterial (**C**) and fungal (**D**) community composition were assessed by Constrained Principal Co-ordinates Analysis (CPCoA). CK: enclosure control; F: ploughing; J: application of *B. thuringiensis* NL-11; C: application of biochar; JC: application of *B. thuringiensis* NL-11 + biochar.

Alpha diversity indices including Shannon, Chao1, and ACE were used to assess the microbial community diversity. Both bacterial and fungal alpha diversity were marginally reduced under all restoration treatments (Figure 6A,B), though non-significant. However, CPCoA indicated significant separation of bacterial (24.14% variance explained) and fungal (21.45% variance explained) communities among treatments (*p* = 0.001). Consistently, all restoration strategies induced distinct beta diversity patterns in both communities (Figure 6C,D). Fungal analysis revealed a distinct separation between the application of *B. thuringiensis* NL-11 and other restoration groups.

**Figure 7:**
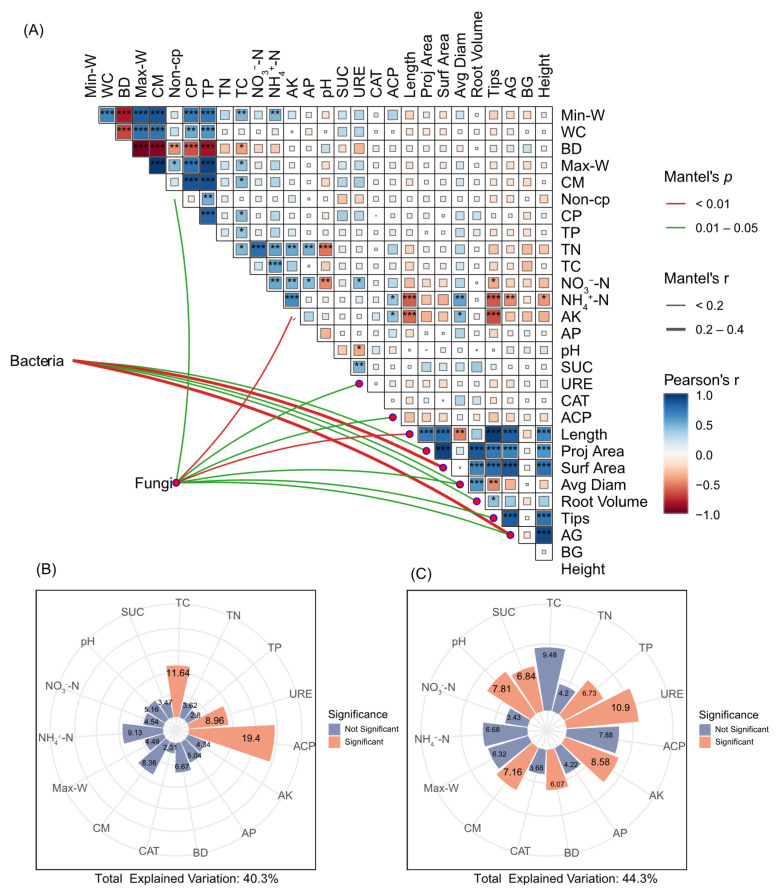
Mantel test shows the relationships between environment factors, *O. japonicus* growth and soil microbial diversity (**A**). The main factors affecting bacterial (**B**) and fungal (**C**) structure are characterized by hierarchical partitioning analysis, the edge colour denotes statistical significance. Pairwise correlations of these variables are shown via a colour gradient, denoting Pearson’s r correlation coefficients. Significance levels are as follows: * *p* < 0.05, ** *p* < 0.01, *** *p* < 0.001. SOC: Organic carbon; AP: Available phosphorus; AK, available potassium; TN: total nitrogen; TC: total carbon; pH value; SUC: sucrase activity; CAT: catalase activity; ACP: acid phosphatase activity; URE: urease activity; Nit-N: nitrate nitrogen; NH_4_^+^-N: ammonium nitrogen; NO_3_^−^-N: nitrate nitrogen; length; proj area: projected area; surf area: surface area; avg diam; root volume; tips; AG: aboveground biomass; BG: underground biomass; Height: plant height.

The Mantel test revealed significant correlations between bacterial community structure and specific plant traits, including root projected area, root average diameter, and root volume (*p* < 0.05); and root surface area with plant aboveground biomass (*p* < 0.01) (Figure 7A). For fungal communities, significant associations were observed with soil non-capillary porosity, soil urease activity, soil acid phosphatase activity, root average diameter, root tips, and plant aboveground biomass (*p* < 0.05), while soil available potassium and root length demonstrated stronger correlations (*p* < 0.01). The different restoration treatments altered soil properties and the beta diversity patterns of associated bacterial and fungal communities.

The hierarchical partitioning analysis in Figure 7B,C indicates that soil variables collectively explained 40.3% of bacterial community variation, identifying acid phosphatase activity as the predominant contributor (19.4%), soil total carbon (11.64%) and soil urease activity (8.96%) were secondary factors. Fungal community structure demonstrated higher explanatory power (44.3%), with soil urease activity representing the foremost regulatory factor (10.9%), succeeded by available potassium (8.58%) and soil pH value (7.81%).

*Proteobacteria*, *Actinobacteriota*, *Acidobacteriota*, *Chloroflexi*, and *Verrucomicrobia* dominated the bacterial phyla (Figures S1 and S2). Differential analyses indicated significant enrichment of *Chloroflexi* and *Verrucomicrobia*, but depletion of *Proteobacteria*, in the JC group relative to CK. At the class level, the J and JC groups showed enriched *Alphaproteobacteria*, *Acidobacteriae*, *Thermoleophilia*, *Actinobacteria*, *Gammaproteobacteria*, *AD3*, *Verrucomicrobiales*. The J group significantly increased *Acidobacteriae* abundance relative to CK, while the JC group elevated *Thermoleophilia* and *AD3*. Within the fungal community, the saprotrophic class *Sordariomycetes* exhibited an elevated relative abundance at the genus level in both the J and JC groups.

## 4. Discussion

The results showed that biochar amendment significantly improved soil physicochemical properties. Its inherently porous architecture enhances aeration, water retention, and nutrient adsorption capacity [30,59], mitigating soil compaction and creating favourable conditions for root proliferation [60,61]. Existing research has indicated that the application of biochar can raise the soil pH slightly [62], thus improving acidic soil through the pH buffering of biochar to provide improved condition for soil restoration and plant growth [63], especially in trampled bare soils. This finding was consistent with our study, where the ploughing and biochar groups were more effective in regulating the soil pH compared with other restoration measures. Intensification in low pH soils leads to alleviation of acid-related retardation of microbial growth and organic matter degradation, leading to large losses of carbon through microbial decomposition [64].

We found that, relative to other soil parameters, soil enzyme activities were particularly responsive to the combined application of *B*. *thuringiensis* NL-11. This enzymatic stimulation reflects enhanced soil biological functionality and nutrient provisioning capacities under microbial–carbon synergies, aligning with documented mechanisms of soil condition improvement [27,65,66]. Previous studies have similarly demonstrated that fertilization may alter specific microbial communities and regulate key functional genes involved in molecular transformations, thereby facilitating microbe-mediated organic matter conversion and enhancing soil biological activity, which provides corroborative support for our findings [67]. CPCoA and between-group variance analyses revealed that the J and JC in a distinct separation from the other restoration groups, indicating treatment-specific restructuring of both fungal and bacterial assemblages [35,68]. Evidence indicates that combined application of biochar and organic fertilizers promotes microbial-driven soil metabolic cycling by enhancing fungal dispersal limitation and modulating microbial activity, thereby reducing nutrient loss [69,70,71].

We observed that restoration treatments differentially affected *O. japonicus* growth and root architecture. Specifically, *B. thuringiensis* NL-11, biochar, and their combined application significantly enhanced plant growth. Regarding root development, while both *B. thuringiensis* NL-11 and its combined treatment with biochar demonstrated superior efficacy in morphometric parameters (root length, surface area, and tip proliferation), a paradoxical reduction in root volume emerged under the combined treatment. Despite exhibiting high branching complexity, the combined treatment yielded disproportionately low root volume, likely reflecting preferential stimulation of lateral roots over primary roots by the microbial inoculant; alternatively, extended biomass accumulation periods may be required [72]. Our findings, which indicate that microbial amendments drive a shift toward fine-root-dominated, acquisitive strategies in *Ophiopogon japonicus*, resonate with the “do-it-yourself” extreme of the collaboration gradient, where plants favour direct resource uptake via high specific root length (SRL) over carbon-intensive symbiotic infrastructures, particularly in degraded soils in which rapid nutrient foraging outweighs structural investment [73,74,75]. Biochar amendment significantly stimulated root length, surface area, and volume in *O. japonicus* relative to CK. Although root tips and fork densities increased under biochar treatment, these enhancements remained lower than those induced by *B. thuringiensis* NL-11 application. Existing research has discovered that the porous structure of biochar possesses a greater adsorption capacity, which enhances soil water and nutrient retention capacities [30]. These improved soil physicochemical properties may also benefit plant root systems to potentially facilitate root tillering and elongation [59]. Particularly toward mitigating the impacts of soil compaction stress on plant growth [60]. This disparity arises from divergent biochemical pathways through which the bacterium and biochar promote root development: the bacterium likely enhances growth via hormonal modulation and elevated root metabolic activity, whereas biochar primarily improves soil aeration, water retention, and nutrient adsorption capacity [76]. We found that bacterial assemblages were predominantly shaped by phosphorus cycling, mediated by acid phosphatase activity, whereas fungal communities were mainly governed by nitrogen metabolism, as indicated by the dominant role of soil urease activity in structuring fungal assemblages [35].

In our study, we observed that the JC group exerted the most pronounced promotive effect on plant growth. However this may be attributed to the intrinsic properties of biochar, which can alter soil nutrient availability and physical structure; on the other hand, biochar also provides nutrients for *B. thuringiensis* NL-11, ensuring its persistence and functionality in the soil [77]. Moreover, biochar particles can serve as microhabitats that enable the longer-term survival of beneficial bacteria, including Bacillus spp., through immobilization on their surfaces; such biochar–microbe formulations improve inoculant persistence and crop performance [77,78]. Together, these factors contributed to the significant restructuring of the microbial community under the JC treatment. Our results showed an increased relative abundance of *Actinobacteriota* and a significant decline in *Proteobacteria* in JC group soils. *Actinobacteriota* are well known for their capacity to decompose recalcitrant organic matter, whereas *Proteobacteria* are typically regarded as copiotrophic microorganisms [79,80]. Within the fungal community, the saprotrophic class *Sordariomycetes* exhibited elevated abundance at the genus level in both the J and JC groups, consistent with its capacity to degrade complex organic substrates [81]. Overall, we found that the C and JC groups favoured microorganisms specialized in the decomposition of organic matter. Unexpectedly, in our study, both the application of strain *B. thuringiensis* NL-11 and its co-application with biochar reduced fungal and bacterial α-diversity (non-significantly), which may be attributed to enhanced competitiveness of specific microbial taxa [24,43,82]. Biochar–microbe formulations enhance soil biological activity, augmenting nutrient micro-cycling and utilization efficiency, thereby promoting plant growth [83,84]. And biochar-microbe associations have been shown to exert synergistic effects: plant growth-promoting bacteria (PGPR) enhance nutrient availability and root development, while biochar improves soil physical properties, structure, and nutrient retention, collectively promoting soil regeneration and increasing ecosystem productivity [17]. Our inference is constrained, on the one hand, by the short duration and use of a single site, which may overlook seasonal and soil-type variability, and on the other hand, by uncertain inoculant dynamics; although biochar can act as an effective carrier enhancing inoculant persistence and colonization, we did not monitor survival [64,85,86]. Future work should use multi-year, multi-site designs and explicitly monitor inoculants to generalize these results.

## 5. Conclusions

This study demonstrates that integrating *B. thuringiensis* NL-11 with biochar carriers exerts synergistic effects on soil microbial community structure, enzyme activities, and plant growth in trampled urban forest soils. Bacterial communities were primarily driven by phosphorus-cycling enzymes, while fungal assemblages were shaped by nitrogen metabolism, with β-diversity analyses indicating a pronounced fungal response to *B. thuringiensis* NL-11 treatment. The combination of microorganisms and biochar enhanced nutrient availability and facilitated spatial resource exploitation, enabling fungi to progressively dominate in nutrient-poor microsites over time. These results not only confirm the effectiveness of microbe–biochar systems for structural and functional rehabilitation of degraded soils but also provide practical guidance for urban forest restoration strategies. Future research should investigate multi-seasonal and multi-site applications, monitor microbial inoculum dynamics, and assess the long-term effects of microbe–biochar interactions on soil function and plant performance to optimize restoration outcomes.

## Data Availability

The obtained sequences were submitted to the NCBI Sequence Read Archive (SRA) under accession number PRJNA1262336.

## Supplementary Materials

The following supporting information can be downloaded at: https://www.mdpi.com/article/10.3390/microorganisms13092004/s1. Figure S1: Composition of bacteria (A) and fungi (B) at the phylum, class, and order levels within the com-munities; Figure S2: Differential abundance analysis across taxonomic ranks; Table S1: Basic properties of biochar; Table S2: Impact trends of soil physicochemical properties under different restoration treatments.
