# Supplementary material for: Synergistic Effects of Biochar and Bacillus thuringiensis NL-11 on Ophiopogon japonicus Growth and Soil Microbial Diversity in Trampled Urban Forest Soils
Source: Microorganisms. 2025 Aug 27;13(9):2004. doi: 10.3390/microorganisms13092004 (PMC12472134; doi:10.3390/microorganisms13092004)
Supplement: Supplementary file 1 [file microorganisms-13-02004-s001.zip › microorganisms-3754413-supplementary.pdf]

Table S1 Basic properties of biochar.

**Raw Material**	**Pyrolysis Temperature**	**Heating Type**	**pH**	**Carbon Content (%)**	**Ash Content (%)**	**Particle Size (mesh)**	**Specific surface area（m²/g)**
Straw	550-600°C	Oxygen-free, Nitrogen Gas Protected Sintering	7-7.5	≥95%	5-10%	100	900-1300

Table S2 Impact trends of soil physicochemical properties under different restoration treatments.

Recovery measures	Mini-moisture content（g/kg）	Bulk density (g/cm³)	Max-moisture content（g/kg）	Capillary moisture content（g/kg）	No capillary porosity /%	Capillary porosity /%	Total porosity /%
CK	259.75±20.39d	1.38±0.13a	309.44±42.81d	280.28±21.49c	3.83±2.72a	38.6±1.65c	42.43±2.11c
F	368.59±56.22bc	1.13±0.11bc	457.2±75.94bc	397.21±40.48b	6.53±4.22a	44.77±0.91b	51.3±3.98b
J	347.96±32.03bcd	1.16±0.08bc	426.71±56.37bc	387.41±53.09b	4.53±1.65a	44.8±3.41b	49.33±2.94b
C	480.11±80.22a	0.96±0.05c	584.9±54.51a	551.52±52.37a	3.2±2.12a	52.93±2.58a	56.13±2.57a
JC	380.14±50.95b	1.05±0.16c	487.31±75.09ab	450.21±58.81b	3.73±1.63a	46.63±1.27b	50.37±1.95b

CK: enclosure control, F: ploughing, J: application of *B. thuringiensis* NL-11, C: application of biochar, JC: application of *B. thuringiensis* NL-11 + biochar. Different letters indicate significant differences between different restoration measures ($P < 0.05$)

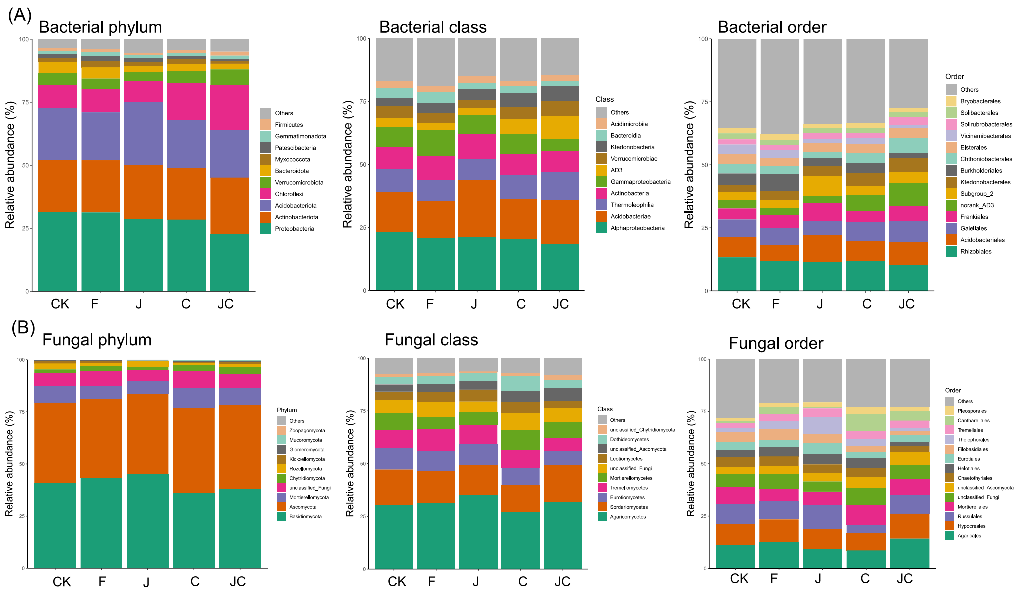

Figure S1 Composition of bacteria (A) and fungi (B) at the phylum, class, and order levels within the communities.

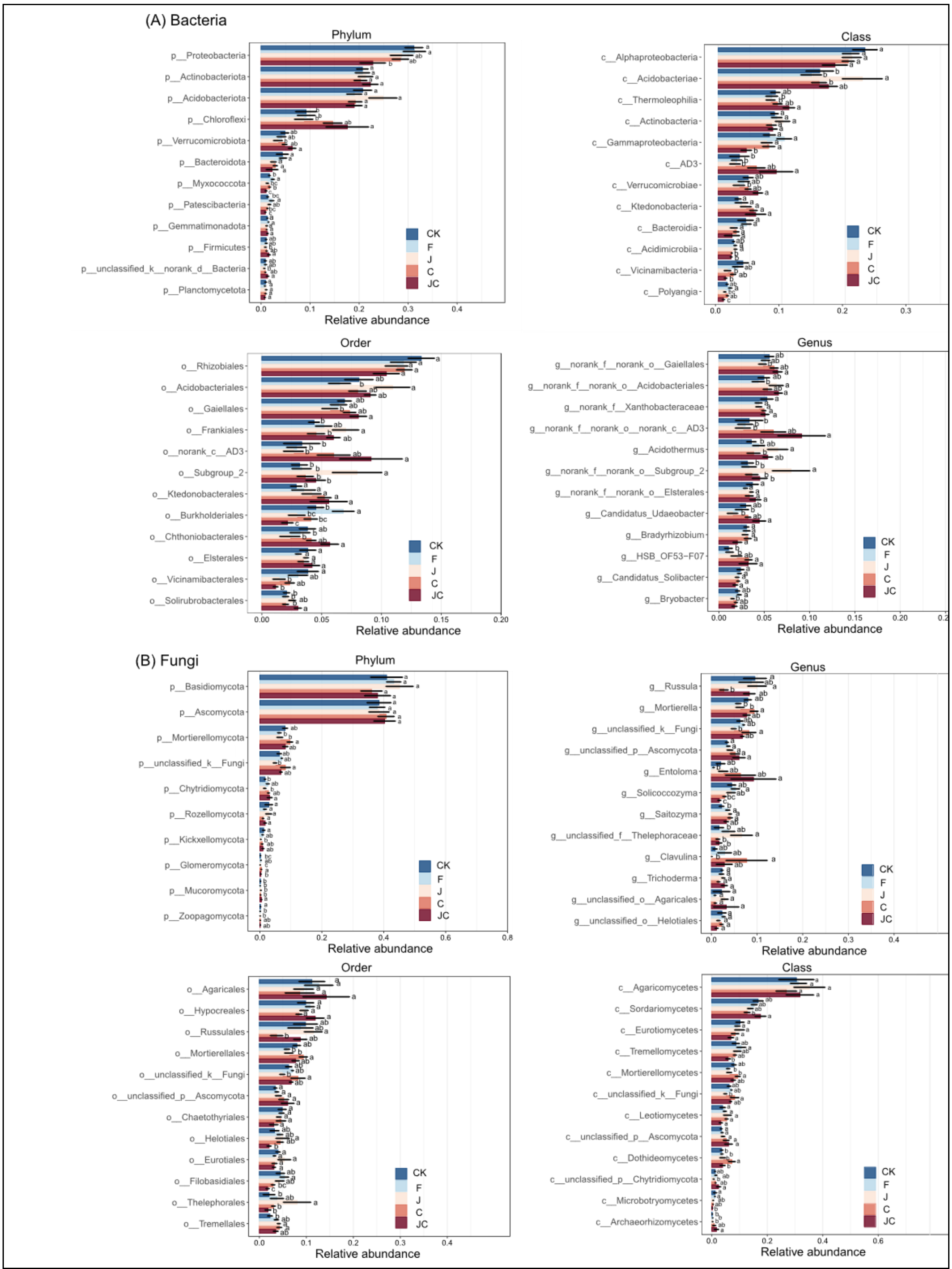

Figure S2 Differential abundance analysis across taxonomic ranks, with node coloration (red) indicating significantly differential taxa (DESeq2 test, Benjamini–Hochberg corrected $P < 0.05$), different letters showed significant differences between different treatments ($P < 0.05$).
